# The epicardial delivery of cardiosphere derived cells or their extracellular vesicles is safe but of limited value in experimental infarction

**DOI:** 10.1038/s41598-021-01728-y

**Published:** 2021-11-12

**Authors:** Verónica Crisóstomo, Claudia Baéz-Diaz, Virginia Blanco-Blázquez, Verónica Álvarez, Esther López-Nieto, Juan Maestre, Antoni Bayes-Genis, Carolina Gálvez-Montón, Javier G. Casado, Francisco M. Sánchez-Margallo

**Affiliations:** 1 Fundación Centro de Cirugía de Mínima Invasión Jesús Usón, Carretera N-521, km 41, 10071 Cáceres, Spain; 2 CIBERCV, Instituto de Salud Carlos III, Madrid, Spain; 3 ICREC Research Group (Insuficiència Cardíaca i REgeneració Cardíaca), Institut d’Investigació en Ciències de la Salut Germans Trias i Pujol, Badalona, Spain; 4 Present Address: Immunology Unit, University of Extremadura, Cáceres, Spain; 5 Present Address: Institute of Molecular Pathology Biomarkers, University of Extremadura, Cáceres, Spain

**Keywords:** Cardiology, Preclinical research

## Abstract

The epicardial administration of therapeutics via the pericardial sac offers an attractive route, since it is minimally invasive and carries no risks of coronary embolization. The aim of this study was to assess viability, safety and effectiveness of cardiosphere-derived cells (CDCs), their extracellular vesicles (EVs) or placebo administered via a mini-thoracotomy 72 h after experimental infarction in swine. The epicardial administration was completed successfully in all cases in a surgery time (knife-to-skin) below 30 min. No significant differences between groups were found in cardiac function parameters evaluated using magnetic resonance imaging before therapy and at the end of the study, despite a trend towards improved function in CDC-treated animals. Moreover, infarct size at 10 weeks was smaller in treated animals, albeit not significantly. Arrhythmia inducibility did not differ between groups. Pathological examination showed no differences, nor were there any pericardial adhesions evidenced in any case 10 weeks after surgery. These results show that the epicardial delivery of CDCs or their EVs is safe and technically easy 3 days after experimental myocardial infarction in swine, but it does not appear to have any beneficial effect on cardiac function. Our results do not support clinical translation of these therapies as implemented in this work.

## Introduction

To this day, the potential of cardiac cell therapy as an ancillary treatment for acute myocardial infarction patients has not been consistently proved. Preclinical and clinical evidences with autologous heart derived products, such as cardiosphere-derived cells (CDCs) were highly promising, but clinical trials using allogeneic cells reported moderate improvement at best^1–6^.

Recent studies have tried to expand cellular therapy indications to the acute setting^2,7–9^, an attractive scenario that could tip the scales towards myocardial salvage/preservation rather than repair/regeneration.

In order to be ready in the acute phase, there are several items to consider. Firstly, therapy should be ready off-the-shelf, which implies that, in the case of cell therapy, cells will need to be allogeneic in origin. The safety of some types of allogeneic cells, such as mesenchymal stem cells (MSCs) or CDCs^10^ has been tested to the scientific community satisfaction now. Allogeneic cells would allow intervention early after myocardial infarction, a must if we want to preserve the tissue and the extracellular matrix (ECM). The ECM, once considered a passive scaffold, is increasingly recognized as playing a dynamic and fundamental role in post-infarction remodelling^11,12^.

CDCs have demonstrated robust safety results, but consistent clinical efficacy has been elusive^3,6^. When administering live therapies such as cells into the post-infarction myocardium, a clear limitation is the ability of the administered cells to survive implantation, let alone engraft in the myocardium. The microenvironment after an infarction is highly hostile, with a myriad deleterious signals such as cytokines, reactive oxygen species, inflammatory cells, cellular debris, etc. precluding survival of cells transplanted into the myocardium early in this process^13^. As a way to bypass this limitation and being able to administer therapy early after infarction, several studies exploring the intrapericardial route have established its feasibility and overall safety post myocardial infarction^7,14–18^, with other studies also exploring antiarrhythmic potential of this route^19^. While the intracoronary route is generally well tolerated, our own studies^20^ proved that it may be harmful when applied on the same day as experimental myocardial induction, despite other groups experiences to the contrary^8,21^. Moreover, early after infarction the existence of no-reflow may compromise cell delivery to the target areas if using the intravascular route beyond the first hour after reperfusion^21^.

Preliminary studies have proved not only that the pericardium is an ideal medium for cell survival^7^, but also that CDCs delivered intrapericardially could potentially exert a beneficial immunomodulatory effect^14^. There is a general shifting in the field towards what has been termed as “cell-less” therapy, using different substances known to be secreted by stem cells, such as extracellular vesicles (EVs), including exosomes^15,22–24^. Intrapericardial EVs have also proved beneficial in the acute myocardial infarction setting^15^. It has been suggested that the immunomodulatory effect that has been reported for CDCs^14^ and replicated with EVs administration^15^ could indeed be linked to the rich content in immune-related proteins and miRNAs that has been described in EVs derived from CDCs^25^. Indeed, our previous studies have demonstrated an immunomodulatory effect of EVs which is reflected by a polarization towards M2 monocytes^15^.

A recent compilation of priorities and challenges in the field points out, among others, the necessity of performing head-to-head comparisons of EVs and their parental cells^11^. In this work, we hypothesize that the administration of CDCs or their EVs into the pericardial sac will allow for longer effect (greater cell survival bypassing the cardiac milieu and the reservoir effect of the pericardial sac), be better tolerated in the acute stage (as it is performed via a minimally invasive surgery without any risk of the administered therapies causing coronary embolization) and it may offer the optimal possibility for the agents to work, since they remain longer onsite, and can therefore secrete paracrine factors responsible for their therapeutic use for a longer period.

In our prior works we have fully characterized the EVs released by CDCs^25^ and the immunomodulatory effects of these cells and vesicles were tested in a porcine model of myocardial infarction^14,15^ but did not look into effectiveness as infarction therapy. The aim of the present study is therefore to perform a head to head comparison of the administration of CDCs or their EVs via the pericardial sac early after myocardial infarction and study their safety and comparative efficacy in terms of cardiac function using clinically relevant models and techniques.

## Methods

### Therapy preparation

#### CDCs isolation culture and characterization

CDCs of swine origin were obtained and characterized as previously described^14^. Briefly, 1–2 g of porcine atrial tissue were minced and digested enzymatically using PBS with 0.2% trypsin (Lonza) and 0.2% collagenase IV (Sigma) in PBS at 37 °C, three times, and then washed with Complete Explant Medium (CEM) and cultured with CEM at 37 °C and 5% CO_2_.

Twenty-one days later, fibroblast-like cells migrating from tissue explants were recovered and transferred to 30 mm poly-D-lysine coated plates with Cardiosphere Growing Medium (CGM). These culture conditions result in the formation of cell clusters (cardiospheres) in suspension from which CDCs separate and migrate. These cells were selected and passed to culture flasks with CGM for expansion (37 °C and 5% CO_2_). At passages 5–10, CDCs were frozen in 90% FBS and 10% dimetilsulfoxide (DMSO), and stored in liquid nitrogen until they were needed. Prior to injection, cells were thawed, centrifuged and resuspended in CGM, filtering them through a 40 µm sieve to assure no cell clusters are injected into the coronary artery. CDCs were then stained with FITC-conjugated porcine monoclonal antibodies against CD29, CD45, CD31, CD90, CD44, SLAI and SLAII, including their isotype controls. Flow cytometric analysis was performed on a FACScalibur cytometer (BD Biosciences) after acquisition of 10^5^ events (Supplementary Fig. 1). Moreover, cells were counted by Trypan blue exclusion, centrifuged again and resuspended in Sodium Chloride 0.9%.to a 6 × 10^6^ cells/mL to a total volume of 5 mL. This solution was supplied to the operating team masked and under sterile conditions.

#### Isolation of EVs from CDCs

EVs were obtained from cell lines cultured at a confluence of 80% at passages 12–15 as reported^25^. Culture medium was replaced by exosome isolation medium (1% insulin-transferrin-selenium in DMEM with 10% Penicillin/Streptomycin). After 4 days, supernatants were collected and subjected to two steps of centrifugation at 4 °C, first at 1000 × g for 10 min, and then 5000 × g for 20 min. The obtained supernatants were then filtered through a 0.22 μm filter to eliminate dead cells and debris, and subsequently ultra-filtered through a 3 kDa MWCO Amicon^®^ Ultra device (Merck-Millipore, MA, USA) to concentrate the EVs. Concentrates were recovered and stored at − 20 °C until use.

For the intrapericardial administrations, protein quantifications were performed by Bradford assay, and the resulting protein concentration was diluted in Sodium Chloride 0.9% (w/v) to 1.832 mg/mL. Finally, 9.16 mg of exosomal proteins were intrapericardially administered in 5 mL of Sodium Chloride 0.9%.

### Animal procedures

**Figure 1:**
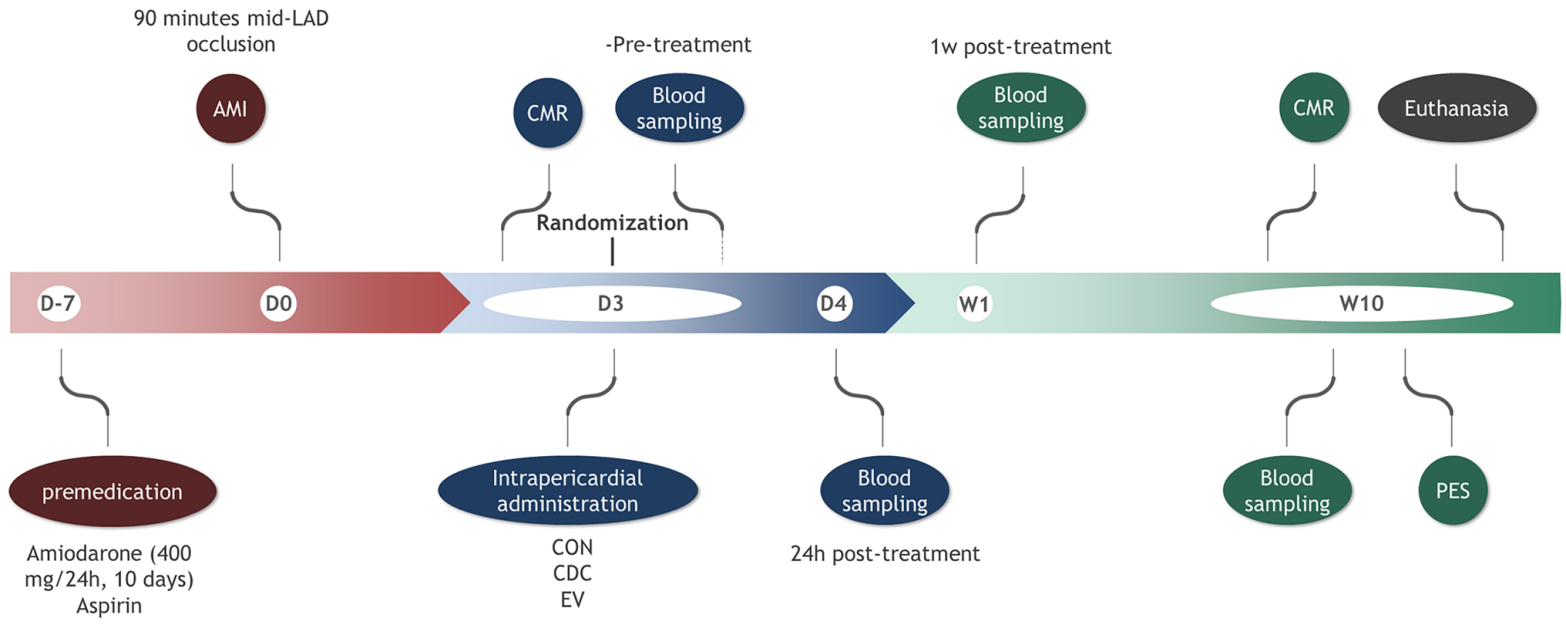
Large animal experimental workflow. Flow chart illustrating the study design in Large White swine. *AMI* acute myocardial infarction, *CON* control group, *CDC* cardiosphere-derived cells group, *EV* extracellular vesicles group, *LAD* left anterior descending coronary artery, *CMR* cardiac magnetic resonance, *PES* programmed electrical stimulation.

All study protocols were approved by the Jesús Usón Minimally Invasive Surgery Centre Animal Care and Use Committee (Ref 018/16) and the Extremadura Regional Government (Exp 20170123-4) and complied fully with Directive 2010/63/EU of the European Parliament on the protection of animals used for scientific purposes. Our animal study is reported in accordance with the ARRIVE Guidelines for reporting experiments involving Animals. Thirty-five female Large White swine were subjected to infarct creation. Cardiac magnetic resonance (CMR) was performed 72 h after infarction, and animals presenting with infarct size > 10% of the left ventricle and ejection fraction < 40% were included in the study and blindly allocated to one of three groups: Control group (CON), CDCs group (CDC) or EVs group (EV). The studies performed are summarised in Fig. 1.

#### Anaesthesia protocol

All procedures were performed under general anaesthesia: animals were premedicated by 20 mg/kg intramuscular ketamine (Ketamidor 100 mg/mL, Richter Pharma AG), induction was achieved with 3 mg/kg intravenous (IV) 1% propofol (Propofol-Lipuro; BBraun) and anaesthetic maintenance performed with inhaled sevoflorane (1.8–2% inspiratory fraction). Endotracheal tubes were connected to a semi closed circular anaesthetic circuit attached to a ventilator (Maquet Flow i) with a fresh gas flow rate of 1 L/min (0.4/0.6 mixture of oxygen and air). Controlled ventilation was established with a tidal volume of 10 mL/kg to obtain normocapnia (with a CO_2_ pressure of 40–45 mmHg). Lidocaine (Lidocaína 2% Braun, BBraun) was administered continuously at a rate of 1 mg/kg/h. Anaesthetic monitoring included cardiovascular and hemodynamic parameters such as: heart rate, electrocardiography, pulse-oximetry and invasive arterial blood pressure.

#### Infarct induction

The infarct induction protocol has been detailed elsewhere^26^. Briefly, a percutaneous transluminal coronary angioplasty (PTCA) balloon catheter (2.5–3.5 mm in diameter. Ryujin plus PTCA dilatation catheter, Terumo, Inc.) was placed immediately distal to the first diagonal branch of the left anterior descending coronary artery via a percutaneous femoral approach. The balloon was inflated to occlude the target artery, and the occlusion maintained for 90 min. Animals were maintained under anaesthesia for 60 min after the balloon was removed to treat any arrhythmias that may occur.

#### Therapy administration

**Figure 2:**
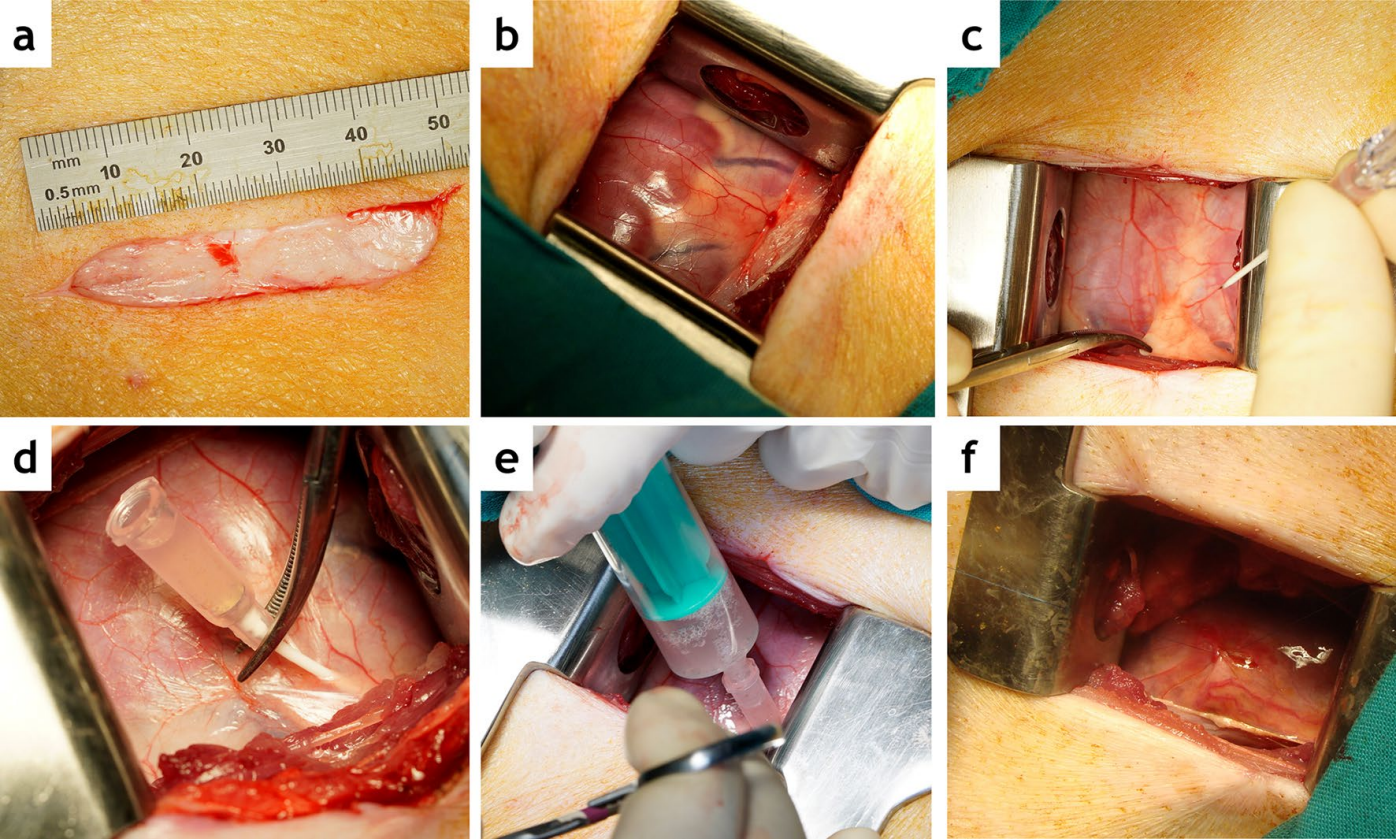
Surgical technique for intrapericardial administration. (**a**) 5 cm long skin incision in the left chest wall. (**b**) The heart is exposed through the mini-thoracotomy performed at the fourth or fifth intercostal space. (**c**) An 18G Abbocath catheter is inserted in the pericardial sac. (**d**) Pericardial fluid filling the hub of the catheter. (**e**) Injection is performed slowly. (**f**) If necessary, a 6/0 polypropylene suture is applied to the pericardial sac.

Three days after infarction, animals were anaesthetized again following the above described protocol, and CMR examinations acquired. Animals complying with the inclusion criteria were then moved to the operating room and placed on the surgical table on the right lateral decubitus. Surgery was performed by a team blinded to group allocation. The left chest wall was surgically prepped and a 5–6 cm long incision made in the fourth or fifth intercostal space to expose the pericardial sac (Fig. 2a, b). A delicate traction was exerted in the pericardium to allow the introduction of an 18G Abbocath catheter within it (Fig. 2c). Whenever feasible, 2–3 mL pericardial fluid were evacuated prior to administering the therapy, to avoid any risk of cardiac tamponade (Fig. 2d). Injection was performed slowly while checking for the appearance of premature ventricular complexes (PVCs) (Fig. 2e). The abbocath was then removed and the pericardium checked for leakage. If necessary, a 6/0 polypropilene suture was applied to the pericardial sac (Fig. 2f). The surgical incision was closed by layers. Animals were allowed to recover from anaesthesia and taken back to the animal housing facility, where post-operative care routinely involved observation, vitals, wound cleaning and antibiotics and analgesia.

#### Follow up

##### Blood sampling and analyses

Blood samples were taken at different times (before treatment, 24 h and one week after treatment, and at euthanasia) and general haematology and biochemistry performed to ensure animal wellbeing. Moreover, cardiac troponin I (cTnI) was also determined at the post-therapy timepoints to rule out additional cardiac damage.

Five animals were randomly chosen from each group to perform cytokine assays. A panel comprising IL-1β, IL-4, IL-6, IL-8, IL-10, IL-12p40, IFN-α, IFN-γ, TFN-α was analysed (Luminex kit: Cytokine&Chemokine 9-Plex Porcine ProcartaPlex™ Panel 1) to check the animals immune and inflammatory state through the study.

##### CMR examinations

CMR studies were acquired 3 days and 10 weeks after infarction, as previously described^2^. Briefly, anesthetized swine were placed inside the magnet (Intera 1.5T, Philips Medical Systems. Best, The Netherlands) in the sternal decubitus, and a dedicated 5 elements cardiac coil was placed around the animals’ chest. Left ventricular function, including end diastolic volume (EDV), end systolic volume (ESV) and ejection fraction (EF) were measured from short axis breath hold gradient echo cine images obtained over the entire left ventricle (LV). Ventricular volumes were normalized by body surface area (BSA), for ease of comparison over time. Infarct size (IS) was computed from short axis images acquired 5–15 min after the injection of 0.2 mmol/kg of a gadolinium-based contrast agent (Gadobutrol. Gadovist 1.1 mmol/L, Bayer Schering Pharma AG) using a breath-hold 3D gradient-echo inversion-recovery sequence. Central dark zones within the area of hyperenhancement were included in the calculations. All calculations were performed by a blinded operator.

#### End study

Immediately after the 10-weeks CMR, animals were transferred to the angio-suite room for cardiac catheterization and programmed electrical stimulation (PES). No lidocaine was used during anaesthesia on the end study day. After obtaining a coronary angiogram, a quadrapolar cathether (Marinr SC Steerable Quadrapolar Catheter, Medtronic) was used to attempt induction of ventricular tachycardia (VT). PES was performed at 3 different cycle lengths with up to 4 extrastimuli (S2-S5), with decreasing coupling intervals in 10 ms until S2 reaches the refractory period or a minimum coupling interval of 200 ms. PES was performed sequentially from the right and left ventricles to assess the possibility that the administered therapies could cause arrhythmogenesis. Once the follow-up was completed, animals were euthanized by a lethal dose of potassium chloride (1–2 mmol/kg) while under deep anaesthesia.

Access to the left thorax was obtained again and the pleura and pericardium were carefully observed for post-surgical changes such as adhesions. The pericardium was opened and hearts explanted. Photographs were taken to document infarct location and size, as well as the existence of any changes to the epicardium that could be caused by the intrapericardial therapy administration. The hearts were then sliced into 10–15 mm thick slices. These slices were stained with a triphenyltetrazolium chloride (TTC) solution, in order to macroscopically assess infarction size and site.

Heart samples obtained from the remote, infarcted and infarct border myocardial zones were fixed in 10% buffered formalin and paraffin-embedded for histological evaluation using haematoxylin–eosin and Masson's trichrome staining.

### Data analysis

Data are presented as means ± standard deviations. Differences between groups were identified and compared using the Kruskal–Wallis and Mann–Whitney U tests, and intragroup comparisons were performed with the Wilcoxon paired samples test. Binary data was studied using chi-square tests. Values of *p* < 0.05 were considered significant. All p values were the results of 2-tailed tests. Calculations were performed using the IBM SPSS Statistics 27 statistical package for Windows.

In order to evaluate the safety of both the surgical approach and the administered therapies, different parameters were considered stratified by the time of observation, as reflected in Table 1.

**Table 1:** Parameters included in safety evaluation.

Period	Safety parameters
During surgery	Surgical time (knife-to-skin)
	Iatrogenic cardiac puncture
	Cardiac arrhythmia
	Haemorrhage
Immediately after surgery	Pleural effusions
	Infections
	Pain (not controlled by routine analgesia)
	Delayed recovery of ambulation
Postoperative period	Post cardiac injury syndrome (PCIS)
	Systemic inflammation (as determined by cytokines assay)
	Haematological and biochemical changes including cardiac markers
	Infection (wound or systemic) or abscess formation
	Fever
End study	Ventricular tachycardia inducibility
	Pericardial adhesions
	Pathology

Clinical efficacy was studied in terms of cardiac function, as determined using CMR, comparing EF, indexed ventricular volumes, infarct sizes and therapeutic effect, defined as the changes seen over time in the calculated parameters (ΔEF, ΔEDVi, ΔESVi, ΔIS).

## Results

A total of 35 animals were subjected to model creation. Of these, 5 animals died during infarction due to refractory malignant arrhythmias (14% of model-related mortality), so that 30 animals were subjected to the 72 h CMR examination. Three animals did not meet the inclusion criteria (infarct size > 10% and LVEF < 40%), and were not included in the study. The remaining animals were allocated to one of the three groups (n = 9 in each group) using an online random number generator (https://www.graphpad.com/quickcalcs/randomize1/) and subjected to surgery by a team blinded to group allocation.

### Safety analyses after CDCs and EVs administration via minithoracotomy in infarcted swine

The intrapericardial administration was completed successfully in all cases in a surgery time (knife-to-skin) below 30 min. Upon accessing the pericardium, accidental myocardial puncture without wall perforation occurred in 4 animals (15%). Digital compression over the puncture site achieved hemostasia in all cases. No cases of VT were seen during surgery.

Daily animal observation in the pens proved that postoperative evolution was uneventful, without signs of infection, undue pain or evidences of pericardial inflammation or post-cardiac injury syndrome (PCIS) ultimately resulting in symptomatic pericarditis.

One animal belonging to the EV group died of unknown causes three weeks after surgery. Necropsy in this animal did not show any treatment-related cardiac or pericardial changes.

No pericardial adhesions were evidenced in any case 10 weeks after surgery.

PES was performed prior to euthanasia in all animals. VT induction was not significantly different between groups (*p* = 0.61), with tachycardia being successfully induced in 1 animal from the control group and one from the CDC group.

### Cytokines

**Figure 3:**
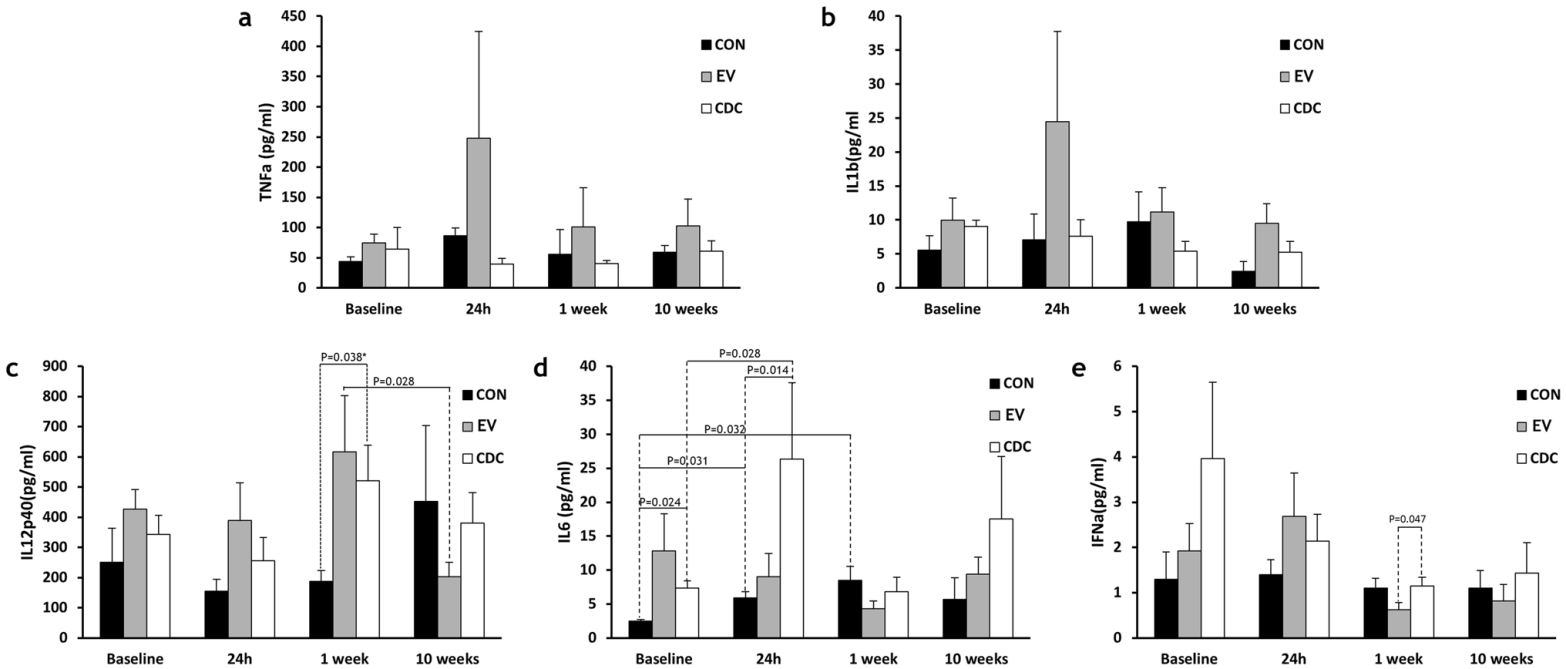
Changes to plasma cytokines detected over time. Measurements of plasma cytokines revealed that four out of nine cytokines (i.e. IFNγ, IL-4, IL-8 and IL-10) were not consistently detected in the samples. The evolution over time of the detected cytokines is shown. *CON* control group, *CDC* cardiosphere-derived cells group, *EV* extracellular vesicles group. (**a**) TFN-α or (**b**) IL-1β levels were not significantly different between groups over time, (**c**) IL12p40 was significantly different between the three groups one week after therapy. *Post-hoc comparison showed these differences to be due to CON versus treated groups (*p* = 0.027, CON vs EV, and *p* = 0.027 CON vs CDC), (**d**) IL-6 showed the greatest change in CDC-treated animals 24 h after therapy and (**e**) IFN-α showed a slight (but significant) difference between CDC and EV one week after treatment.

Of the assayed cytokines, IL-4, IL-8, IL-10 and IFN-γ were not consistently detected in the samples, being generally below the detection threshold, so they were not included in the analyses. The remaining cytokines levels are shown in Fig. 3. No significant changes over time or between groups were seen in either TFN-α or IL-1β, although in both cases the levels measured at 24 h after surgery were higher in the EV group. TFN-α values were 247.72 ± 354.92 pg/mL in EV versus 86.07 ± 22.08 pg/mL and 39.76 ± 20.91 pg/mL in CON and CDC groups, respectively (Fig. 3a). Similarly, IL-1β was 24.44 ± 29.85 pg/mL in EV versus 7.09 ± 7.58 pg/mL and 7.6 ± 5.47 pg/mL in CON and CDC groups, respectively (Fig. 3b).

Differences in IL12p40 were seen between the 3 groups one week after therapy, as shown in Fig. 3c (*p* = 0.038), with CON animals having significantly lower values than both EV and CDC groups (188.6 ± 70.5 pg/mL vs 617.7 ± 414.3 pg/mL and 521 ± 264.8 pg/mL, respectively). IL12p40 levels decreased significantly from one to ten weeks after therapy in EV-treated animals (617.7 ± 414.3 pg/mL to 202.9 ± 107.5 pg/mL, *p* = 0.028).

IL-6 evolution is shown in Fig. 3d. Significant increases over time in this cytokine were seen in CON, from pre-treatment to 24 h (*p* = 0.031) and 1 week (*p* = 0.032) later (from 2.48 ± 0.4 to 5.9 ± 21.94 pg/mL and 8.5 ± 4.18 pg/mL). At one day after treatment, IL-6 values measured in CDC-treated animals were significantly increased from those determined prior to therapy administration (from 7.4 ± 2.22 to 26.33 ± 25.24 pg/mL, *p* = 0.028). Intergroups comparisons did not show significant differences between the three groups at any timepoint, although IL-6 levels were higher in CDC compared to CON animals at both pre-treatment (when levels were 7.4 ± 2.22 pg/mL in CDC vs 2.48 ± 0.4 pg/mL in CON, *p* = 0.024) and 24 h later (26.33 ± 25.24 pg/mL vs 5.9 ± 1.94 pg/mL, *p* = 0.014).

Finally, IFN-α did not change significantly over time in any group (Fig. 3e). Its values at 1 week were slightly but significantly (*p* = 0.047) higher in CDC compared to EV (1.15 ± 0.42 pg/mL vs 0.63 ± 0.33 pg/mL).

### Cardiac function

**Figure 4:**
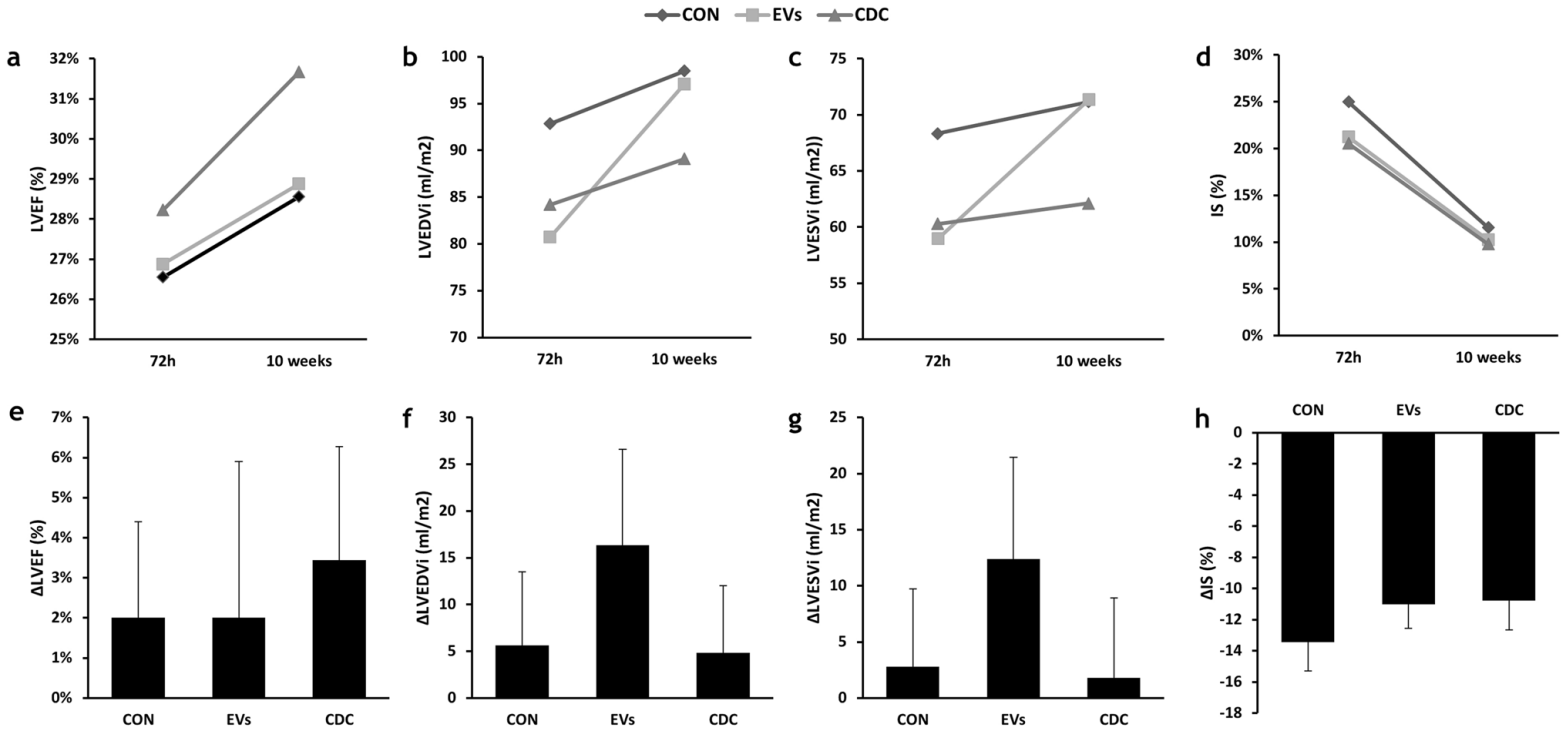
Evolution of cardiac function parameters after intrapericardial CDC, EV or Control administration in infarcted swine. (**a**–**d**) Changes over time in cardiac function parameters as measured with cardiac magnetic resonance (CMR) for the three experimental groups (CON, EV and CDC). (**a**) Evolution of left ventricular ejection fraction (LVEF). (**b**) End diastolic volume indexed to body surface area (EDVi). (**c**) End systolic volume indexed to body surface area (ESVi). (**d**) Evolution of infarct size. (**e**–**h**) Treatment effects (defined as the difference between pre-injection and 10-week values). (**e**) Effects on left ventricular ejection fraction (LVEF). (**f**) End diastolic volume indexed to body surface area (EDVi). (**g**) End systolic volume indexed to body surface area (ESVi). (**h**) Effects on infarct size.

Cardiac function parameters as measured with CMR are reflected in Fig. 4 and Table 2. No significant differences between groups were found in CMR-derived parameters at the end of the study, despite a trend towards improved cardiac function (greater EF and smaller ventricular volumes) in CDC-treated animals.

**Table 2:** Main cardiac parameters calculated from magnetic resonance exams performed throughout the study.

Groups	CON		EV		CDC	
	Baseline preinjection	10 weeks	Baseline preinjection	10 weeks	Baseline preinjection	10 weeks
LVEF (%)	27 ± 3	29 ± 7	27 ± 4	29 ± 13	28 ± 5	32 ± 8
EDVi (mL/m^2^)	93 ± 17	98 ± 19	81 ± 11	97 ± 29	84 ± 9	89 ± 20
ESVi (mL/m^2^)	68 ± 13	71 ± 19	59 ± 9	71 ± 31	60 ± 8	62 ± 21
Infarct size (%)	25 ± 7	12 ± 3	21 ± 5	10 ± 2	21 ± 3	10 ± 3
Cardiac output (L/min)	1.8 ± 0.3	2.6 ± 0.4	1.3 ± 0.3	2.4 ± 0.9	1.8 ± 0.4	2.8 ± 0.6
Beats per minute	93 ± 12	90 ± 7	86 ± 12	102 ± 17	95 ± 12	95 ± 16
Δ LVEF (%)	n/a	2 ± 7	n/a	2 ± 11	n/a	3 ± 8
Δ EDVi (mL/m^2^)	n/a	6 ± 24	n/a	16 ± 29	n/a	5 ± 21
Δ ESVi (mL/m^2^)	n/a	3 ± 21	n/a	12 ± 26	n/a	2 ± 21
Δ Infarct size (%)	n/a	− 13 ± 6	n/a	− 11 ± 4	n/a	− 11 ± 2

Data presented as mean ± standard deviation.

*LVEF* left ventricular ejection fraction, *EDVi* end diastolic volume indexed to body surface area, *ESVi* end systolic volume indexed to body surface area.

Infarct area is expressed as % of the left ventricle. *n/a* not applicable.

The evolution of the studied cardiac function parameters was similar in all three groups, with small increases in EF (Fig. 4a) from pre-injection to 10 weeks, slightly but not significantly greater in CDC-treated animals. Ventricular volumes were not significantly different between groups either, with all groups showing dilatations over time, more pronounced (albeit not significantly so) in the EV group. Treatment effects (defined as the difference between pre-injection and 10-week values), also not significantly different between groups, are shown in Fig. 4e–h.

### Pathology results

**Figure 5:**
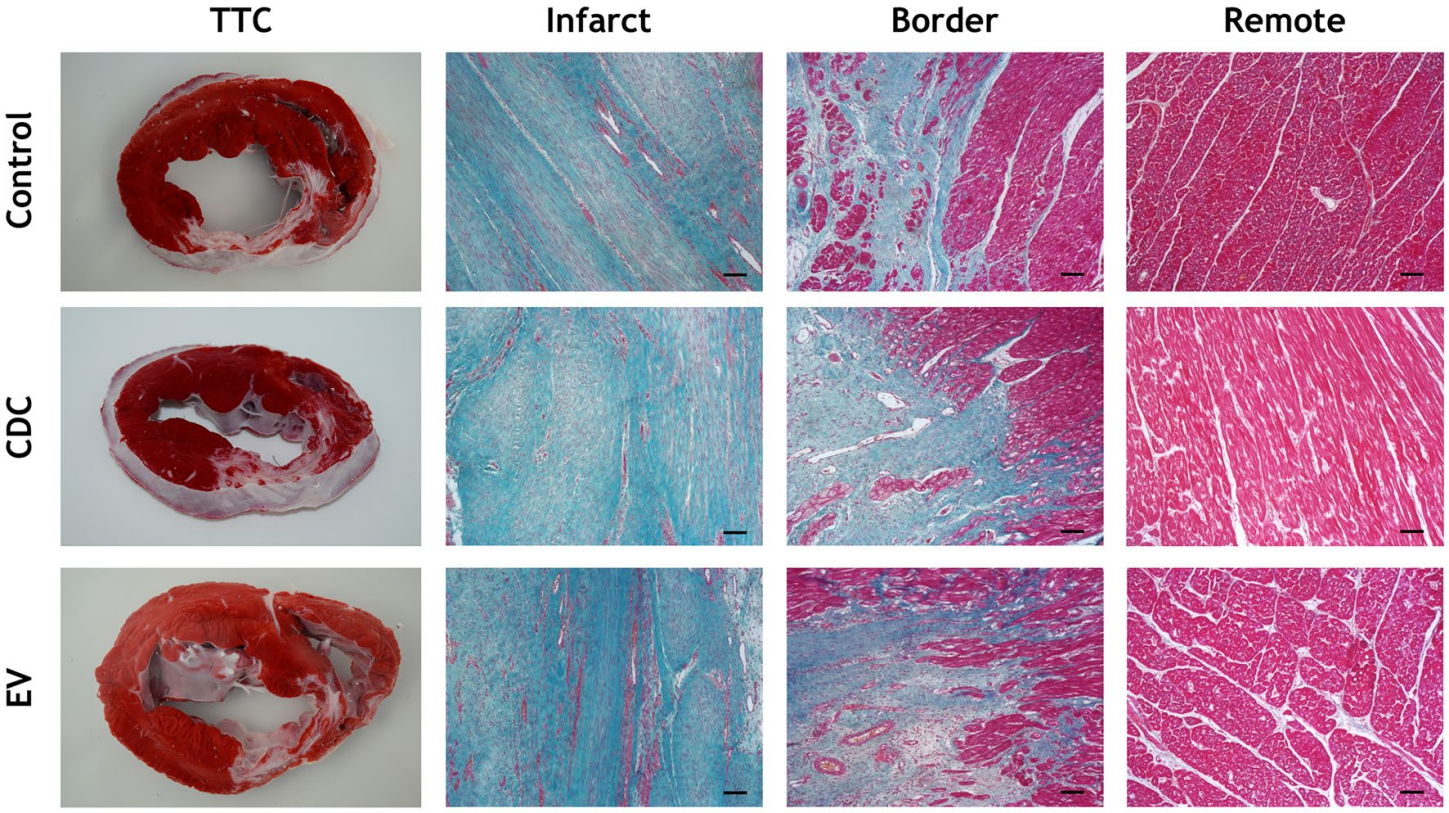
Histopathological studies. Photographs of heart slices stained with a triphenyltetrazolium chloride (TTC) solution showing macroscopically infarction size and site of CON, CDC and EV animals. Representative images of Masson’s trichrome staining of infarct, border and remote myocardial zones of CON, CDC and EV animals. Scale bar = 50 µm.

On 5 µm-thick paraffin sections, haematoxylin–eosin and Masson’s trichrome (Fig. 5) descriptive staining showed no evident anatomopathological differences between groups neither in remote, nor in infarct and border zones.

## Discussion

The present study analysed safety and efficacy of early delivery of heart-derived therapeutics into the pericardial sac via a mini-thoracotomy. The use of clinically relevant imaging techniques and a large animal model ensured that our results could be translatable to the clinical scenario. The main findings of this study are that the surgical technique is very safe and easy to perform; however, we failed to detect any significant clinical improvement after long term follow-up in the included animals. In spite of early indications of immunomodulation capacity, we could not demonstrate long term functional benefits.

As a delivery route, the pericardial sac offers potential advantages over the intracoronary, intramyocardial or transendocardial routes, in that it may increase therapy concentration locally with limited systemic exposure, it carries no risk of vascular embolization and is not hindered by lack of coronary patency. For these reasons, it has been used as a promising access to administer different therapies, including antiarrhythmic^27–29^, angiogenic^30^ or cell therapy^7,14,16,31,32^. Similarly, different approaches to access the pericardial sac have been described, including percutaneous, transatrial o with the use of dedicated devices or techniques^33–36^. Pericardial access is routinely performed in the electrophysiology lab under imaging guidance for epicardial VT ablation^36^. The LARIAT procedure for left atrial appendage exclusion is also an important therapeutic strategy that includes pericardial access. Percutaneous accesses to the pericardium, however, are not devoid of complications, many of which can be minimized or completely avoided with direct visualization as used in this study. Vascular injury to adjacent blood vessels, visceral injury or right ventricular puncture or tear commonly associated to the subxiphoid method^37^ are prevented using this approach. Other common complications, such as pericarditis, pericardial effusion or tamponade were not seen with the mini-thoracotomy approach either, although they cannot be completely ruled out. Moreover, it obviates the need for fluoroscopic guidance, and allows the identification of pericardial adhesions or changes prior to puncture. Therefore, considering the ease of performance and excellent safety profile of the mini-thoracotomy approach, it could be used in when prior post-cardiac injury syndrome is suspected to avoid puncture risks. Surgical site has been recognized as a major player in postoperative pulmonary risk factors, with thoracic surgery representing the second most important procedure related risk contemplated in the guidelines^38^. Hence the safety of this procedure, while intuitively good, needed to be established. Our results show this procedure to be safe and easy to implement, in absence of any adverse effect in this experimental setting.

As a further safety evaluation, the cytokine profiles were studied over time in a subset of animals from the 3 groups. We could not consistently detect IL-4, IL-8, IL-10 and IFN-γ, which could be related to the detection limit of commercially available swine immune reagents. The analysis allowed us to quantify TFN-α, IL-1β, IL12p40, IL-6 and IFN-α, although statistically significant differences could only be observed in IL12p40 and IL-6. This absence of changes to typically proinflammatory cytokines, such as TFN-α, IL-1β and IFN-α supports the above-mentioned high safety profile. On the other hand, the significant increase of IL-6 after myocardial infarction has been previously described by several authors^39^ and seems to be associated with the risk of future myocardial infarction^40^. In the case of IL-6, our results reflect an inflammatory response which is detected in CON and CDC groups, but not in the EV group. We hypothesize that the immunomodulatory activity of EVs which has been found to be involved in the regulation of the IL6/IL6R axis^25^, may counteract the inherent inflammatory damage linked to the infarction and subsequent early surgical procedure. Regarding IL12p40, the variability and changes observed among study groups and different time points hinder the interpretation of the results. While IL12 heterodimer is generally proinflammatory, the p40 subunit has been reported to act as IL12 antagonist, because it competes with its binding to the IL12 receptor (IL12Rβ1), thus presenting pleiotropic effects^41^. The increase in IL12p40 in both treated groups compared to control one week after therapy may then reflect a modulation of IL12-driven inflammation rather than a proinflammatory effect.

Unfortunately, we could not demonstrate any consistent functional benefit to either CDCs or EVs administration via the pericardial space. Exosomes derived from CDCs have been previously described to recapitulate the effects of CDCs in a porcine model similar to ours^42^, but only when administered transendocardially using the NOGA system as opposed to intracoronary delivery. Prior experimental results, both from our own lab^7,14,15,25^ and others^16,18,32^ encouraged the pericardial approach to regenerative cardiac therapy. Initially, the pericardial fluid has been thoroughly characterized and proven as an ideal medium for allogeneic MSCs survival^7^, although xenogeneic human MSCs delivered into the pericardial space in swine only survived when they were protected within alginate microcapsules^16^. Treating cardiospheres with pericardial fluid obtained from infarcted rats enhanced biological activity without compromising cell viability and resulted in improved ventricular function in treated rats^32^. Prior works with both autologous endothelial progenitor cells^18^ and allogeneic CDCs^14^ have shown that after being delivered to the pericardial sac these cells migrate into the myocardium. Moreover, CDCs engraftment to the damage tissue has also been demonstrated in cardiomyopathies^43^ in part mediated by adhesion surface markers^44^. In an acute immunological study^15^, our group has shown that intrapericardial EVs but not CDCs promote M2 monocyte polarization, a finding that, considering the proangiogenic and anti-inflammatory capacity of said monocytes also paved the way for the present work. Lastly, we have recently evaluated the immune-related genes expression in the infarcted myocardium following intrapericardial secretomes delivery, and found a significant impact on the expression of immune-related genes that confirms the immunomodulatory potential of intrapericardially-delivered secretomes^25^. These studies, however promising, did not follow up the experimental subjects in the long term, with the longest follow-up reported being 4 weeks in both small^32^ and large^14^ animals.

Recent results from the ALLSTAR clinical trial^6^, pointed to disease modifying activity of CDCs that nonetheless did not decrease IS compared to placebo. Similarly, we did not detect significant improvements in cardiac function, but immunomodulation could be identified in the EV group, as reflected by the cytokines results. While this lack of significant improvement in functional parameters could be related to the small sample size (a limitation frequently encountered in large animal studies for ethical considerations), it is rather disappointing. Still, translational studies such as this one are needed to confirm or disprove hypothesis that could well go out into the clinic, and avoid negative or indifferent results in clinical trials with all that entails. In this sense, failure to publish negative or indifferent results, known as publication bias, has been identified as a source of major overestimation of efficacy (by about 30%) in animal studies of stroke^45^. It is very clear that unbiased science requires publication of all kind of results. The above mentioned clinical trial, ALLSTAR^6^ was discontinued due to “futility”; since the interim analysis performed at 6 months after intervention revealed very low probability of it reaching its primary efficacy endpoint, namely a 15% decrease in infarct size at 12 months. This clinical trial and the present study, while using the same cell type, have little else in common, since the ALLSTAR patients in the recent myocardial infarction cohort received the therapy around 2 months after the index event; which certainly represents a very different scenario from the acute myocardial infarction we are studying in this experimental work. Indeed, the authors recognize that their study does not address the role of intracoronary CDCs infusion in the acute myocardial infarction. Our objective was therefore to study an altogether different scenario if with the same cells. Our results, unfortunately, are very similar to those reported for this clinical trial. This could contribute to prevent wasted efforts and to facilitate production of novel hypotheses.

Regarding IS evolution, it decreased similarly in all three groups. We have previously seen this same time-related effect^2,20,26^. A possible explanation can be found looking at the natural course of ischemia/reperfusion injury and the method used for measuring myocardial function. On the one hand, and while CMR is considered the gold standard for quantifying acute and chronic myocardial infarction, it is known that in the early stages of MI, infarct size can be overestimated by the presence of edema fluid and cellular components, such as hemorrhage and inflammation, which can acutely increase infarct volume by as much as 25%. As this edema and inflammation vanishes and necrotic myocardium is replaced by scar tissue over time, the infarcted area shrinks^46^. This is mostly due to increased extracellular volumes in the acute setting, that will be normalized so that no difference is seen at end study between CMR- and ex vivo TTC-measured infarct sizes^47^.

Similarly, and closely related to this, 3 days after infarction there is still a high level of myocardial stunning, a transient ischemia-related ventricular dysfunction, also associated with prolonged biochemical abnormalities that may take days to resolve following initial resolution of ischemia. Hence the apparent improvement seen in these parameters, independent of the delivered therapy. While we think that the time of therapy represents a distinct opportunity to exploit the potential of the cells, it is true that at the same time the infarct damage is still evolving and, as previously reported, is expected to shrink naturally as edema resolves and fibrosis replaces muscle, causing wall thinning and remodeling. Thus LV wall thinning would also account for a certain amount of apparent decrease in scar size measured as a percentage of the LV.

As shown in Fig. 5, CDCs outperformed EVs in all measured parameters, which could be related to the cells capability to react to environmental signals^25^ and secrete a variable response depending on the host need. For instance, when in a proinflammatory environment CDCs have been reported to respond by immunomodulatory factors, such as miRNAs and proteins. EVs, on the other hand, would have a predetermined capability depending on their parent cells and their environment upon EVs secretion.

This lack of response to intrapericardial EVs is actually in line with prior works by Gallet et al.^42,48^, in which only the intramyocardial administration of EVs yielded significant differences in IS and EF preservation in a swine model compared to intracoronary delivery. Using bioluminescence tracking of far-red labeled exosomes, the authors demonstrated that exosomes retention was much higher in when administered intramyocardially, thus pointing that EVs washout could be responsible for the difference. While it is true that the intrapericardial administration is not subject to vascular washout, prior studies have also expressed the concern that lymphatic clearance needs to be taken into account^16^, since it has been reported that complete pericardial fluid renewal takes place in sheep every 5–7 h^49^, and even in some species there are small pores in the parietal pericardium. We did not, however, conduct any serial study to see the permanence of either EVs or CDCs in the pericardial fluid in the present work. We have previously shown a permanence of cells 7 days after administration using Y-chromosome amplification^7^.

Similar results have been described by others, with early positive results, including also M2 macrophage polarization not conferring longer term benefits in terms of EF or ventricular volumes, albeit increased perfusion in the infarcted zone was seen in adipose tissue derived-MSCs‐treated animals^9^. It is possible that the mechanisms triggered by the therapy administration in this study, as in our own, are short-lived and therefore insufficient to achieve clinically meaningful results. In order to harness and strengthen the action of cell-based therapies, many strategies have been proposed, such as using biomaterials for the administration, modifying the cells with gene therapy, increasing survival by immune-protecting the cells, etc. In a paradigm shift, repeated administration has been advocated^50^. If the lack of beneficial effects seen in this study is indeed due to short-lived action of the administered therapeutics, repeating the dose could definitely improve results and greatly impact future regeneration strategies.

A limitation of all animal studies lies in the need to be careful when extrapolating results to the clinical scenario. While swine are the preferred species for their similarity to human cardiac anatomy and coronary flow, the use of healthy young animals could restrict the applicability of the results, since typically myocardial infarction patients are elderly and suffer from comorbidities, and thus their response to any intervention is influenced by a number of factors that we could not reproduce in this experimental study.

In conclusion, and while the epicardial administration via the pericardial injection of 30 × 10^6^ CDCs or their EVs is safe and technically easy 3 days after experimental myocardial infarction in the porcine model, it does not appear to have any beneficial effect on cardiac function, despite early indication of immunomodulatory capability associated to the EVs. Our results do not support clinical translation of these therapies as implemented in this work for cardiac regeneration, although the mini-thoracotomy as administration route may be worthy of further studies for different applications.

## Supplementary Information

Supplementary Figure 1.
