# Supplementary material for: The epicardial delivery of cardiosphere derived cells or their extracellular vesicles is safe but of limited value in experimental infarction
Source: Sci Rep. 2021 Nov 12;11:22155. doi: 10.1038/s41598-021-01728-y (PMC8590017; doi:10.1038/s41598-021-01728-y)
Supplement: Supplementary file 1 — Supplementary Figure 1. [file 41598_2021_1728_MOESM1_ESM.pdf]

Supplementary information

The epicardial delivery of Cardiosphere Derived Cells or their extracellular vesicles is safe but of limited value in experimental infarction.

Verónica Crisóstomo[1,2], Claudia Baéz-Diaz[1,2], Virginia Blanco-Blazquez[1,2], Verónica Álvarez[1], Esther Lopez-Nieto[1], Juan Maestre[1,2], Antoni Bayes-Genis[2,3], Carolina Galvez-Monton[2,3], Javier G. Casado[1,2], Francisco M. Sánchez-Margallo[1,2]*

1.- Fundación Centro de Cirugía de Mínima Invasión Jesús Usón, Cáceres, Spain
2.- CIBERCV, Instituto de Salud Carlos III, Madrid, Spain
3.- ICREC Research Group (Insuficiència Cardíaca i REgeneració Cardíaca), Institut d'Investigació en Ciències de la Salut Germans Trias i Pujol, Badalona, Spain.

Supplementary figure 1

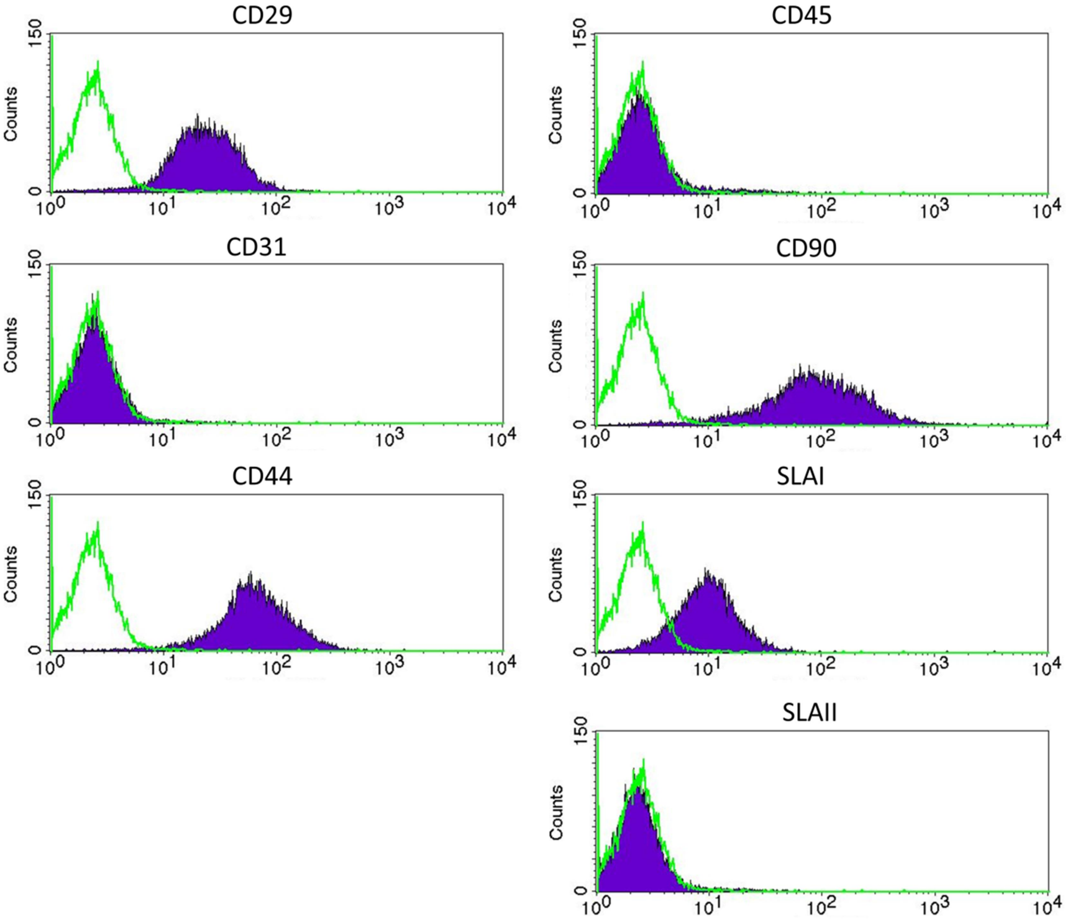

Supplementary Figure 1: Phenotypic analysis of CDCs by flow cytometry. Representative histograms together with the expression levels are shown. Green lines show isotype control and purple fill histograms show the expression level of cell surface markers represented as Mean Relative Fluorescence Intensity.
